# Monolayer Graphitic Carbon Nitride as Metal-Free Catalyst with Enhanced Performance in Photo- and Electro-Catalysis

**DOI:** 10.1007/s40820-022-00794-9

**Published:** 2022-02-03

**Authors:** Huiyan Piao, Goeun Choi, Xiaoyan Jin, Seong-Ju Hwang, Young Jae Song, Sung-Pyo Cho, Jin-Ho Choy

**Affiliations:** 1 Intelligent Nanohybrid Materials Laboratory (INML), Institute of Tissue Regeneration Engineering (ITREN), Dankook University, Cheonan, 31116 Republic of Korea; 2 College of Science and Technology, Dankook University, Cheonan, 31116 Republic of Korea; 3 Department of Nanobiomedical Science and BK21 PLUS NBM Global Research Center for Regenerative Medicine, Dankook University, Cheonan, 31116 Republic of Korea; 4 Department of Materials Science and Engineering, College of Engineering, Yonsei University, Seoul, 03722 Republic of Korea; 5 SKKU Advanced Institute of Nanotechnology (SAINT), Sungkyunkwan University (SKKU), Suwon, 440-746 Republic of Korea; 6 Department of Nano Engineering, Sungkyunkwan University (SKKU), Suwon, 440-746 Republic of Korea; 7 National Center for Inter-University Research Facilities (NCIRF), Seoul National University, Seoul, 08826 Republic of Korea; 8 Graphene Research Center, Advanced Institute of Convergence Technology, Suwon, 16229 Republic of Korea; 9 Department of Pre-Medical Course, College of Medicine, Dankook University, Cheonan, 31116 Republic of Korea; 10 Tokyo Tech World Research Hub Initiative (WRHI), Institute of Innovative Research, Tokyo Institute of Technology, Yokohama, 226-8503 Japan

**Keywords:** Graphitic carbon nitride, Monolayer, Atomic image, Electro- and photo-catalysis

## Abstract

**Highlights:**

- The g-C_3_N_4_ monolayer in the perfect 2D limit was successfully realized, for the first time, by the well-defined chemical strategy based on the bottom-up process.
- The most striking evidence was made from Cs–high resolution transmission electron microscopy measurements by observing directly the atomic structure of g-C_3_N_4_ unit cell, which was again supported by the corresponding high resolution transmission electron microscopy image simulation results.
- We demonstrated that the newly prepared g-C_3_N_4_ monolayer showed outstanding photocatalytic activity for H_2_O_2_ generation as well as excellent electrocatalytic activity for oxygen reduction reaction.

**Abstract:**

The exfoliation of bulk graphitic carbon nitride (g-C_3_N_4_) into monolayer has been intensively studied to induce maximum surface area for fundamental studies, but ended in failure to realize chemically and physically well-defined monolayer of g-C_3_N_4_ mostly due to the difficulty in reducing the layer thickness down to an atomic level. It has, therefore, remained as a challenging issue in two-dimensional (2D) chemistry and physics communities. In this study, an “atomic monolayer of g-C_3_N_4_ with perfect two-dimensional limit” was successfully prepared by the chemically well-defined two-step routes. The atomically resolved monolayer of g-C_3_N_4_ was also confirmed by spectroscopic and microscopic analyses. In addition, the experimental Cs-HRTEM image was collected, for the first time, which was in excellent agreement with the theoretically simulated; the evidence of monolayer of g-C_3_N_4_ in the perfect 2D limit becomes now clear from the HRTEM image of orderly hexagonal symmetry with a cavity formed by encirclement of three adjacent heptazine units. Compared to bulk g-C_3_N_4_, the present g-C_3_N_4_ monolayer showed significantly higher photocatalytic generation of H_2_O_2_ and H_2_, and electrocatalytic oxygen reduction reaction. In addition, its photocatalytic efficiency for H_2_O_2_ production was found to be the best for any known g-C_3_N_4_ nanomaterials, underscoring the remarkable advantage of monolayer formation in optimizing the catalyst performance of g-C_3_N_4_.
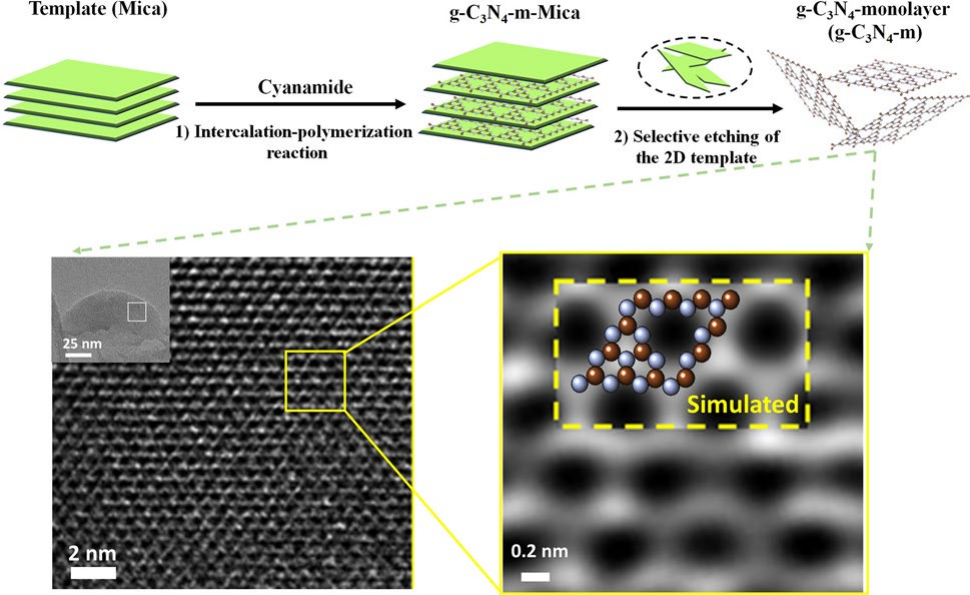

**Supplementary Information:**

The online version contains supplementary material available at 10.1007/s40820-022-00794-9.

## Introduction

Since the pioneering works on graphene were first reported [1–4], many studies with van der Waals (vdW) engineering have yielded functional materials with unprecedented properties. In particular, materials with two-dimensional (2D) structure have been scientifically-important playgrounds in condensed matter chemistry and physics, in part because they were considered as useful building blocks for tunable electronic properties in manifold applications. Therefore, various metallic, insulating and semiconducting 2D materials, *e.g*., graphene, h-boron nitride (h-BN), transition metal oxides (TMOs) and transition metal dichalcogenides (TMDs), have been proposed to be applicable as stackable layers for integration into devices where a variety of pseudo-particles, such as plasmons and excitons, can be generated [5–7]. Recently, the genome of material properties was investigated theoretically based on ab initio calculations of vdW heterostructures as well as thick incommensurable ones [8]. This kind of theoretical works have been extended through massive collaborations using artificial intelligence (AI) databases to facilitate high-throughput material exploration [8].

Moreover, the availability of the additional degrees of synthetic freedom allows the juxtaposition of additional 2D building blocks to generate new material properties such as photocatalytic and electrocatalytic properties. Photocatalysts and electrocatalysts have attracted much attention because of their crucial roles in many renewable energy technologies like photoelectrochemical cells, fuel cells and metal –O_2_ batteries during the past decade [9–11]. The cost for catalyst materials, however, is the most critical hurdle in the industry and has motivated to develop a new route to use alternatives to precious metals. The graphitic carbon nitride (g-C_3_N_4_) in single atom thickness has been, therefore, suggested to be an ideal candidate to overcome such a hurdle, since this semiconducting 2D material boasts outstanding versatility as a metal-free catalyst for various energy-related reactions [12, 13]. Despite widespread interest, it has been remained as a challenging task to identify a chemically well-defined and economically viable route to genuine g-C_3_N_4_ monolayers (described as g-C_3_N_4_–m hereafter) for various applications in the near future. There were some challenging physicochemical routes to nanosheets such as the exfoliation of bulk g-C_3_N_4_ (g-C_3_N_4_–b hereafter) into 2D nano-platelets with several layers to induce high surface area (*e.g*., liquid exfoliation [14], thermal oxidation [15], chemical exfoliation [16, 17]), but such top-down processes gave rise to the formation of nanolayers only, and even they were not chemically and crystallographically well-defined (Table S1). To dates, no report has been found to confirm pure monolayer of g-C_3_N_4_ [18–22]. Therefore, it is crucial to develop new synthetic routes based on creative chemical strategy to well-defined monolayer of g-C_3_N_4_. When a facile way of preparing g-C_3_N_4_–m is realized, g-C_3_N_4_–m will be applied to diverse photo- and electro-catalysis researches due to its enhanced specific surface area, eventually resulting in a great improvement of catalytic-active sites. Furthermore, potentially unprecedented and unique materials could be created by assembling them together with other 2D building blocks along the crystallographic c-axis based on van der Waals engineering. Such new 1:1 type hybrid with heterostructure could have various physical and chemical characteristics because of the synergy effect between those different 2D materials.

**Fig. 1:**
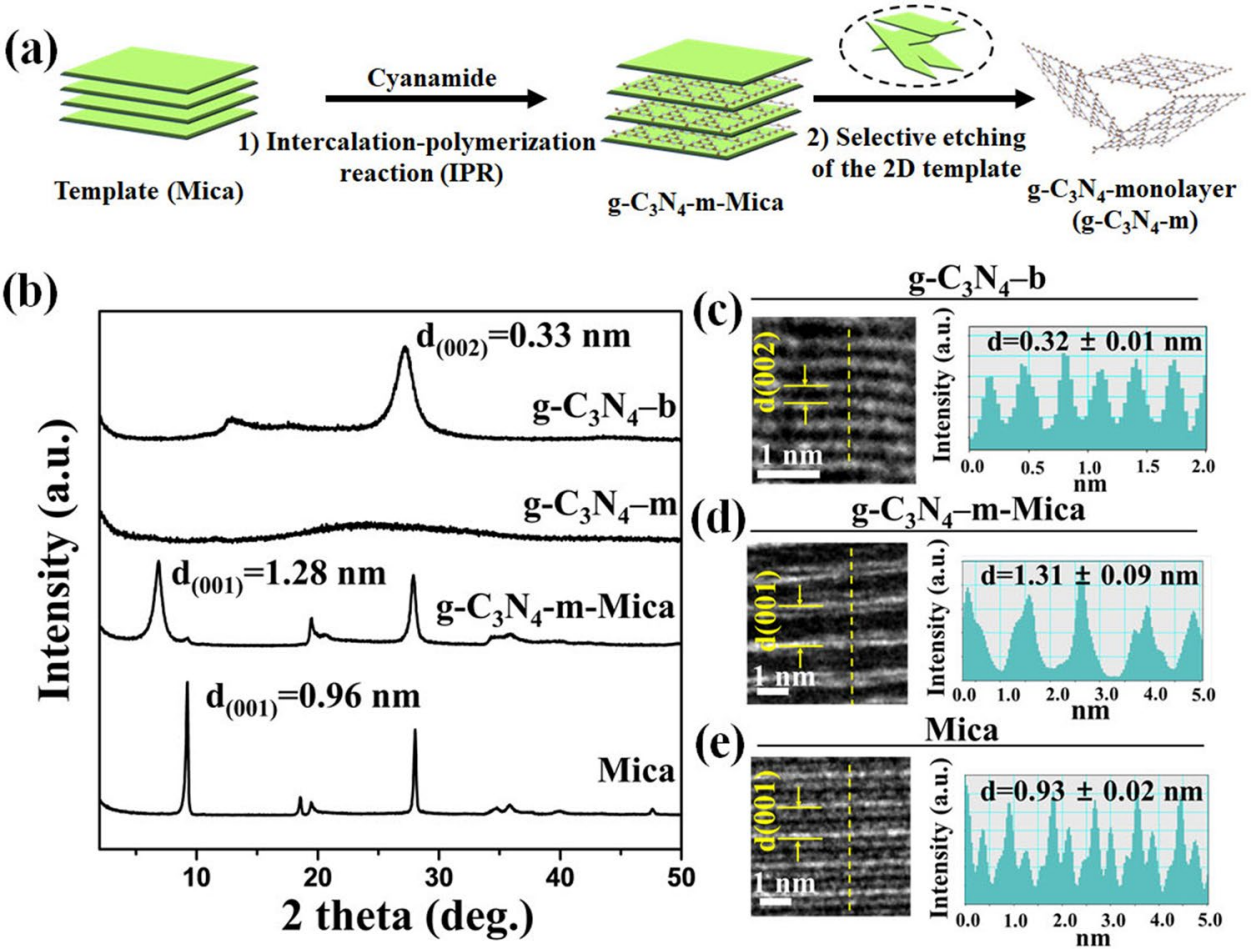
**a** Schematic diagram for the synthesis of g-C_3_N_4_–m by using the 2D Mica template. **b** XRD patterns of Mica, g-C_3_N_4_–m–Mica, g-C_3_N_4_–m and g-C_3_N_4_–b. **c–e** Cross-sectional HRTEM images and photometric intensity profiles along the yellow dashed lines for g-C_3_N_4_–b, g-C_3_N_4_–m–Mica and Mica

In the present study, g-C_3_N_4_–m in the perfect 2D limit was successfully prepared by the chemically well-defined bottom-up approach involving two-step processes: (1) at first, cyanamide precursor molecules were intercalated into 2D Mica template to form cyanamide-Mica hybrid, and thus confined cyanamide in the inlayer space of Mica was subjected to thermal polymerization reaction (designated as the intercalated polymerization reaction, IPR), and (2) finally the selective etching was made to separate g-C_3_N_4_–m only from 2D template as described in Fig. 1a. In the first step, the precursor (cyanamide in this work) was intercalated into the interlayer space of 2D template (Mica in this work) to form cyanamide-Mica hybrid, which was then transformed to g-C_3_N_4_–m–Mica hybrid upon heating at 550 °C by an in-situ polycondensation reaction. In this two dimensionally confined nanospace of Mica, the product tri-s-triazine units were bridged only in lateral direction through single nitrogen atoms periodically to form g-C_3_N_4_–m. From thus prepared g-C_3_N_4_–m–Mica hybrid, well-defined g-C_3_N_4_–m monolayers could be separated by etching Mica template selectively in an acid solution of NH_4_HF_2_ and HCl. Since the g-C_3_N_4_–m monolayers were found to be chemically very stable, they remained undissolved, but suspended in the mother liquid. The formation of g-C_3_N_4_–m with atomically resolved monolayers was confirmed by spectroscopic and microscopic analyses, those which were in excellent agreement with the theoretically simulated one. It is worthy to note here in advance that the g-C_3_N_4_–m monolayer shows unusually high efficiency for photocatalytic H_2_O_2_ generation, underscoring the remarkable advantage of monolayer formation in exploring high-performance g-C_3_N_4_-based catalyst materials.

## Experimental Section

### Materials

Na-fluorine mica (denoted as Mica), with chemical formula of Na_0.7_Mg_2.65_Si_4_O_10_F_2_ and cation exchange capacity (CEC) of 120 meq./100 g, was purchased from CO-OP Chemical Co. Ltd (Japan). Cyanamide (CH_2_N_2_) was purchased from Alfa Aesar Chemical Co. Ltd. (Korea).

### Preparation of g-C_3_N_4_-m

The g-C_3_N_4_–m incorporated in the Mica was prepared by conventional solid-state intercalation reaction. First, cyanamide intercalated Mica (cyanamide-Mica nanohybrid) was synthesized by mixing and grinding cyanamide and Mica with ratio of Mica: cyanamide = 4: 1, and reacting the mixture at 100 °C for 3 h. To polymerize the cyanamide intercalated in Mica, the cyanamide–Mica nanohybrid was calcined in a sealed Pyrex ampoule at 550 °C for 4 h. Finally, the g-C_3_N_4_–m was extracted out from g-C_3_N_4_–m–Mica hybrid by dissolving Mica template simply in NH_4_HF_2_ (4 M) at 0 °C for 4 h. Then, the magnesium fluoride was obtained as an impurity from reaction of NH_4_HF_2_ with Mica. The impurities fluorinated possibly on g-C_3_N_4_–m also were cleaned out by treating with HCl (2 M) at 80 °C for 1 h. The sample was filtered out from an impurity solution, again washed with deionized water, and finally centrifuged to separate the monolayer suspension from the precipitate. The synthetic yield of chemically well-defined g-C_3_N_4_–m was around 52%. On the other hand, the g-C_3_N_4_–b was synthesized by heating the cyanamide at 550 °C for 4 h with the heating rate of 3 °C min^–1^.

### Characterization

The powder X-ray diffraction (XRD) analyses for all the samples were carried out by using a Rigaku diffractometer with Ni-filtered Cu-K radiation (*λ* = 1.5418 Å) operated at 40 kV and 30 mA. The UV–vis diffuse reflectance spectra (DRS) were obtained by using JASCO V-550 (JASCO, Japan) spectrophotometer installed with an integrating sphere, where the BaSO_4_ plate was used as a baseline and the data were converted to Kubelka–Munk (K-M) functions. The photoluminescence (PL) spectra were obtained using fluorescence spectrophotometer (Perkin-Elmer, LS55, USA) with an excitation wavelength at 273 nm. For Fourier transfer infrared (FT-IR) spectroscopic analysis, the bulk carbon nitride, g-C_3_N_4_–b, was prepared on the standard KBr disk substrate, and the monolayer one, g-C_3_N_4_–m, was coated on the standard ZnSe window. In addition, JASCO FT-IR 6100 spectrometer (JASCO, Japan) was used to collect mid infrared data. The X-ray photoelectron spectroscopy (XPS) study was made with a monochromatic Al X-ray source (K-alpha, Thermo VG, UK). To measure the thickness of the g-C_3_N_4_–m, atomic force microscope (AFM) in tapping mode (Veeco, Digital Instrument Dimension 3100, USA) was used. Zeta potential was recorded on a Zetasizer Nano ZEN3600 (Malvern Instruments Ltd., UK). High resolution transmission electron microscopy (HRTEM) and spherical aberration (Cs) corrected-high resolution transmission electron microscopy (Cs-HRTEM) images were taken using a JEM ARM-200F microscope (Image Cs-corrector, JEOL, JAPAN) at 80 kV. The Cs-HRTEM images were obtained with a fast and sensitive 16 Megapixel CMOS camera (OneView camera, GATAN, US), enabling to study atomic scale details by very low-dose and high contrast imaging for beam sensitive samples and low atomic number elements. To minimize electron beam damage, Cs-HRTEM investigations for g-C_3_N_4_–m was carried out using low-dose less than about 80 electrons Å^−2^, which was calculated from the measured one (~ 0.002 nA) using a Faraday cup on the fluorescent screen (in the absence of specimen). No changes in image detail arising from electron beam damage were detected while observing the magnified images. High-angle annular dark-field-scanning transmission electron microscopy (HAADF-STEM) and electron energy loss spectroscopy (EELS) analyses were also carried out with a JEM ARM-200F microscope (Probe Cs-corrector), where an EELS detector (965 GIF Quantum ER, GATAN, US) was equipped and operated at 80 kV.

### Cs-HRTEM Image Simulation

The multi-slice simulation [23] for Cs-HRTEM images was performed using the commercial MACTEMPAS (Total Resolution LLC, US) program. In this simulation, the Cs value was set to − 0.034 mm, due to the fact that if Cs = 0, the phase contrast of very thin film becomes zero at just the point of focus (Gaussian focus) [24], and the other ones were adjusted according to our experimental conditions, and the value of a crystal thickness (1 nm) and the defocus value of a 6 nm steps in the range from − 54–300 nm. The Digital Micrograph software GMS 3.2 (Gatan Inc., USA) was applied for recording/processing the images, including Fourier filtering. Furthermore, the filtered Cs-HRTEM images presented in this study were reconstructed using a Wiener Filter, implemented in a commercial software package (Quantitative HAADF, HREM Research Ltd. JAPAN), to remove artifacts and noise in the original images.

### Photocatalytic Activity Measurement

The evolution of the photocatalytic performance of g-C_3_N_4_ upon the monolayer formation was examined for the evolution of H_2_O_2_ and H_2_ under the illumination of visible light. The UV and IR components from Xe lamp (300 W, Newport) were removed by employing both the optical cutoff filter (*λ* > 420 nm) and water filter, respectively. For the H_2_O_2_ evolution test, 50 mg of photocatalyst was dispersed in 100 mL of aqueous methanolic solution (5.0 vol.%) acting as a hole scavenger. The amount of H_2_O_2_ generated was determined by colorimetry, as reported previously [25]. Prior to the H_2_ generation test, the Pt particles (3.0 wt.%) were deposited as co-catalyst on the photocatalyst materials. The amount of H_2_ gas evolved was evaluated with gas chromatography. 100 mL of aqueous triethanolamine solution (10 vol.%) was utilized as a hole scavenger for 50 mg of photocatalyst.

### Electrocatalytic Activity Measurement

The electrocatalytic activities of the present materials for oxygen reduction reaction (ORR) were measured at room temperature using a standard three electrode electrochemical cell controlled by an IVIUM workstation. A glassy carbon (GC) electrode, a Pt wire, and a saturated calomel electrode (SCE) were utilized as working, counter and reference electrodes, respectively. The O_2_-bubbled 0.1 M KOH solution was used as an electrolyte. The ORR polarization curves were collected by employing a RRDE-3A (ALS Co.) as a rotator with a rotating speed of 1600 rpm. For the preparation of ink solution, 3.2 mg of electrocatalyst, 0.8 mg of Vulcan-XC72R and 20 μL of 5 wt.% Nafion solution were dispersed in 2.0 mL of mixed solvent of Milli-Q water and 2-propanol (4/1, vol/vol) and then subjected to sonication for 1 h, yielding the homogenous electrocatalyst ink. The obtained ink solution (10 μL) was dropped on a GC electrode (3 mm diameter, ALS Co.). The potentials were converted to reversible hydrogen electrode (RHE) scale with following equation: E(RHE) = E(SCE) + 1.012 V. The electrochemical impedance spectroscopy (EIS) data were collected with IVIUM analyzer in the frequency range of 0.1–100,000 Hz at open circuit voltage (OCV) and 0.6 V (vs. RHE).

## Results and Discussion

### Powder XRD, HRTEM, AFM and Zeta Potential Analyses

The crystal structures of the g-C_3_N_4_–m–Mica hybrid and the g-C_3_N_4_–m extracted from the hybrid were studied by XRD and HRTEM. According to the XRD patterns in Fig. 1b, the basal spacing (0.96 nm) of Mica was expanded to 1.28 nm upon the formation of g-C_3_N_4_–m in the interlayer space of Mica. Its gallery height of 0.32 nm (1.28–0.96 nm) should be the same as the thickness of g-C_3_N_4_ monolayer (0.33 nm). The cross-sectional HRTEM images and their photometric intensity profiles were also in good agreement with the XRD data, as demonstrated in Fig. 1c–e. The basal spacings along the crystallographic c-axis were determined to be 0.32 ± 0.01 nm for g-C_3_N_4_–b, 1.31 ± 0.02 nm for g-C_3_N_4_–m–Mica and 0.93 ± 0.09 nm for the intact Mica, respectively. In addition, the well-developed (00 l) XRD reflections in layered compounds can always observed not only for the nanolayered but also for the bulk, the multi-layered. But no (00 l) peak could be observed for the monolayered (g-C_3_N_4_–m) due to the absence of stacking along the crystallographic c-axis (Fig. 1b).

**Fig. 2:**
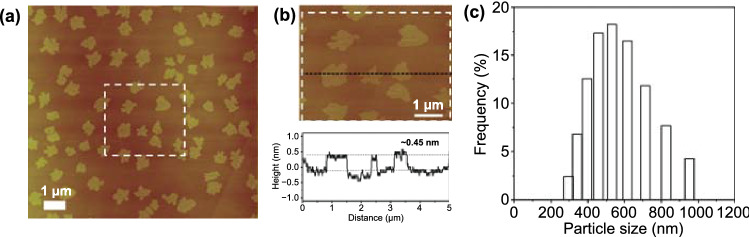
AFM image of g-C_3_N_4_–m. **a** The g-C_3_N_4_–m monolayers dispersed on the freshly-cleaved muscovite mica substrate. **b** The thickness of g-C_3_N_4_–m monolayer flakes, and the height profile along the black dashed line. **c** The histogram for the size distribution of g-C_3_N_4_–m

To synthesize g-C_3_N_4_–m, the g-C_3_N_4_–m–Mica hybrid was dissolved in an acidic solution, where the Mica template was selectively etched out with a formation of colloidal suspension of g-C_3_N_4_–m. The chemically stable g-C_3_N_4_–m monolayer particles were extracted from the acidic solution. The colloidal particles of g-C_3_N_4_–m in an aqueous solution were determined to be positively charged with a zeta potential of + 37 mV, which was found to be very stable for several weeks (Fig. S1). As shown in Fig. 2a, AFM images were recorded in a typical tapping mode for g-C_3_N_4_–m deposited on a freshly-cleaved muscovite mica surface by spin-coating. The monolayer flakes were well dispersed on the substrate and their topographical heights were determined to be ~ 0.45 nm on average (Fig. 2b), which was consistent with the theoretical thickness of monolayer (0.33 nm) [26]. Figure 2c shows a histogram of the size distributions of g-C_3_N_4_–m estimated from AFM images, indicating their lateral sizes of 400–800 nm. Based on XRD and AFM results, we came up with a conclusion that the monolayer of g-C_3_N_4_ was indeed realized for the first time.

### Cs-HRTEM Analysis

**Fig. 3:**
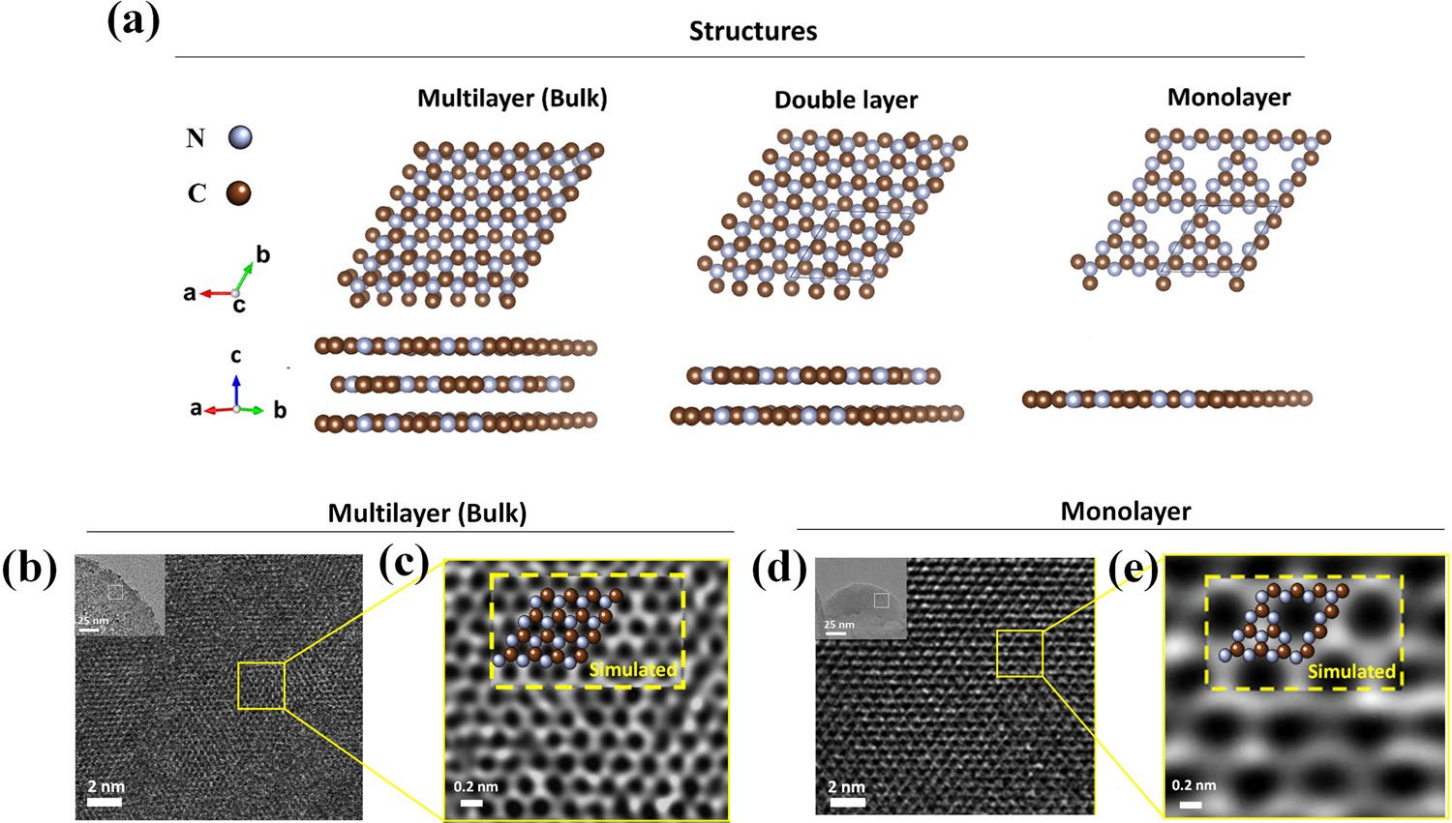
Experimentally observed and theoretically simulated atomic structures for g-C_3_N_4_–b and g-C_3_N_4_–m. **a** Schematic presentation of multilayer (bulk), double layer and monolayer structures of g-C_3_N_4_. **b, d** Cs-HRTEM images of g-C_3_N_4_–b and g-C_3_N_4_–m, respectively. The insets are low-magnification TEM images. **c, e** Wiener-filtered Cs-HRTEM images experimentally observed, which are magnified from the yellow box in b and d. The insets are the images theoretically simulated (yellow dashed box) with the atomic model overlaid of g-C_3_N_4_–b and g-C_3_N_4_–m

The g-C_3_N_4_–m in the perfect 2D limit was further investigated by low dose, Cs-HRTEM. As the electron illumination has to be stronger for its higher magnification, the electron irradiation damage might be inevitable to get high resolution TEM images [27]. HRTEM has been widely applied to study 2D soft materials such as TMD (MoS_2_, WS_2_), graphene and *h*-BN, because they are robust materials with planar bonding groups [6, 28]. In contrast to such 2D nanosheets, however, the atomic configurations of g-C_3_N_4_–m exhibit two unique features that make them more susceptible to electron damage: (1) they contain two distinct light elements (carbon and nitrogen), and (2) they are structurally assembled from repeated tri-s-triazine (C_6_N_7_) units, bridged through single nitrogen atoms. Periodic triangular vacancies within planar domains of monolayer g-C_3_N_4_, different from the double layered and the multilayered, result in a low density of *in-plane* bonds. However, the double layered g-C_3_N_4_ has a staggered and graphite-like AB arrangement with one set of tri-s-triazine units from the first layer superimposed all the way on the top of the voids of the second layer [29]. The g-C_3_N_4_–b phase is also constructed by the ABAB… stacking to form multi-layers without any large triangular vacancies (Fig. 3a). Such a low atomic density in *in-plane* of g-C_3_N_4_–m with periodic vacancies might be the reason why no one was able to observe its monolayer image by TEM. It is surely due to the fact that the monolayer g-C_3_N_4_ is too fragile to withstand the high irradiation energy, even though various attempts were made to reduce irradiation damages by lowering exposure time, electron current, electron energy, etc. [30].

In order to surmount electron beam damage for such a fragile g-C_3_N_4_–m, as mentioned above, we employed Cs-HRTEM instrumentation at the very low-dose of 80 kV, and used a Gatan OneView 4 K camera optimized for both sensitivity and speed. No damages in HRTEM images in both the samples, g-C_3_N_4_–m and g-C_3_N_4_–b, were observed in Fig. 3b–e (additional Cs-HRTEM images are also shown in Fig. S2). As demonstrated in Fig. 3c, e, the enlarged Cs-HRTEM images were obtained from the selected area in Fig. 3b, d (yellow colored box) using Wiener filtering that was applied with commercial software (Quantitative HAADF, HREM Research Ltd. JAPAN), respectively. As can be seen clearly from Fig. 3c, e, a simulated Cs-HRTEM image (inset image) was overlapped on an experimentally observed one, which was calculated by the multi-slice method [23] using the commercial program (MACTEMPAS, Total Resolution LLC, US) with the atomic model overlaid. In this simulation, the tri-s-triazine-based structure model determined by Kroke et al*.* [31] was used to obtain the *in-plane* and the surface-to-normal coordinates on the basis of density-functional theory (DFT), because this structure is the most stable configuration among all the allotropes of g-C_3_N_4_. The simulation parameters were set under our experimental conditions, including a crystal thickness of 1 nm and a defocus (△*f*) value of a step of 6 nm in the range from − 54–300 nm (Figs. S3–S4); first, in the case of mutilayer structure for g-C_3_N_4_–b, its Cs-HRTEM image (Figs. 3b and S5a, b) and the corresponding selected area electron diffraction (SAED) pattern (Fig. S5c, d) clearly demonstrate the honeycomb and hexagonal structure of g-C_3_N_4._ As shown in Fig. 3c, the present Cs-HRTEM image turned out to be quite similar to the graphene one due to the ABAB stacking more than two layers, as well consistent with the simulated one (more Cs-HRTEM images for g-C_3_N_4_–b can be found under different defocus (△*f* = 0 nm) conditions as shown in Fig. S6). In addition, the image for g-C_3_N_4_–b was clearly different from that for g-C_3_N_4_–m as shown in Fig. 3d, e, where one can find periodically ordered bright white dots with triangular arrangement of vacancy in a hexagonal structure model as shown in Fig. 3e. This was in good agreement with the simulated image in the inset.

**Fig. 4:**
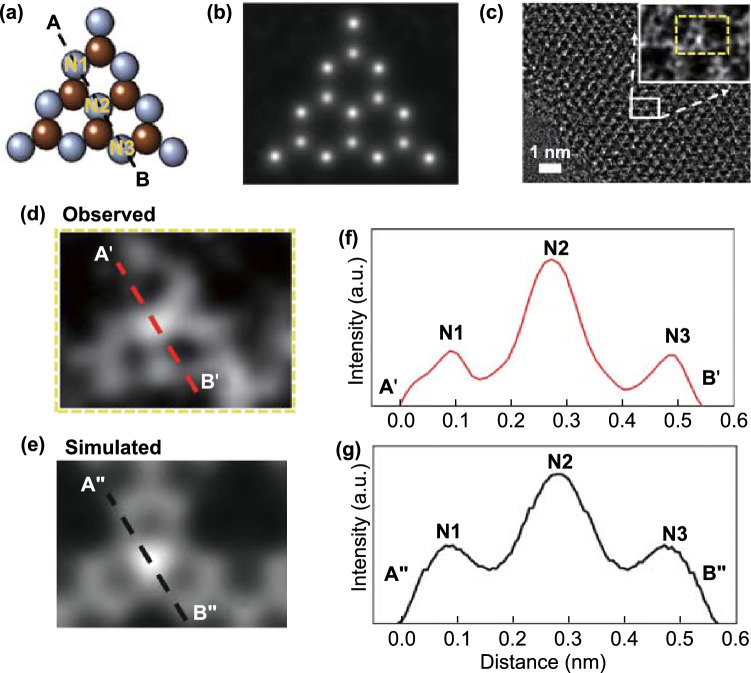
Experimentally observed and theoretically simulated atomic structures of g-C_3_N_4_–m. **a** A structure model and **b** an exit wave function. **c** Wiener-filtered Cs-HRTEM image of g-C_3_N_4_–m. **d** A Cs-HRTEM image magnified from the yellow-dashed box in c, inset. **e** A corresponding simulation image for a tri-s-triazine unit. **f, g** Cross-sectional profiles on the dashed line in d and e. The dashed line of A–B in a corresponds to the dashed lines of A’(A’’)-B’(B’’) in **f** and **g**

In order to further assess atomic arrangements of the monolayer sample, we also performed the through-focal series of simulated images to assist in interpreting experimental Cs-HRTEM images. As shown in Fig. 4a–c, the present Cs-HRTEM images is that the bright-contrast peaks were appeared on the center of every tri-s-triazine unit, where a nitrogen atom was located. From the selected area *d* in Fig. 4c (yellow-dashed box), a magnified Cs-HRTEM image in Fig. 4d was taken, where aromatic rings of C and N were discernible with a strong contrast peak from the nitrogen atom at the triazine center. This observed image was also in good agreement with the simulated one in Fig. 4e. The parameters for this simulation were all the same as the previously used, but with the only exception, the amount of defocus was set to △*f* = 50 nm (see Fig. S4 for more detail in the Supporting Information). According to the detailed evaluation based on the through-focal series simulation, one can find again the periodically ordered bright white dots with triangular cavity in the hexagonal structure model, which was well consistent with the structure model shown in Fig. S4a. The identity of tri-s-triazine unit was further confirmed by the line profile analysis. The line profiles, as shown in Fig. 4f, g, were obtained from intensity scans between *A’(A’’)* and *B’(B’’)* in Fig. 4d, e, where the lines through the *A’(A’’)* and *B’(B’’)* were crossing three different nitrogen atoms (N1, N2 and N3) in the tri-s-triazine unit cell as shown in Fig. 4a. The simulation intensity pattern with defocus value of 50 nm also match well with observed one. It was, therefore, concluded that the monolayer prepared in this study was a perfect atomic monolayer, and quite consistent with the results of tri-s-triazine building blocks calculated by Kroke et al. [31].

### EELS and FT-IR Analyses

**Fig. 5:**
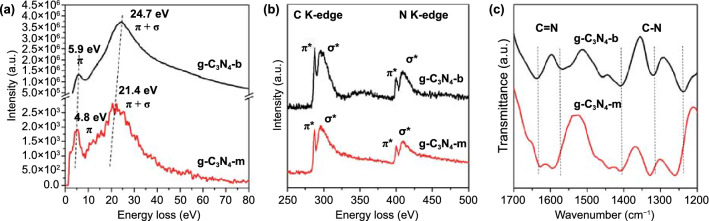
EELS and FT-IR spectra for g-C_3_N_4_–b and g-C_3_N_4_–m. **a** Low-loss and **b** core-loss range spectra of EELS for both g-C_3_N_4_–b and g-C_3_N_4_–m, respectively. The EELS spectra were normalized with respect to the integrated intensity of the negative energy-loss part of zero loss peak (ZLPs). **c** FT-IR spectra of g-C_3_N_4_-b and g-C_3_N_4_–m in the range of the C–N and C = N bonds

In order to further assess the electronic states for the present g-C_3_N_4_–m and g-C_3_N_4_–b were also probed by EELS analysis (corresponding annular dark-field (ADF) images in Fig. S7). According to the low-loss EELS spectra (Fig. 5a), two major absorption peaks were observed at 4.8 and 21.4 eV in the low-loss spectrum from g-C_3_N_4_–m, due to the *π* and *π* + *σ* plasmons, respectively. The same two bands were also consistently appeared from the bulk g-C_3_N_4_–b, but with the stronger intensity and the high-energy shift by 1.1 eV for the *π* plasmon, and by 3.3 eV for the *π* + *σ* one, compared to those from g-C_3_N_4_–m. Such a change in the low-loss EELS spectrum is very similar to that reported in the boron nitride monolayer by Coleman et al. [6]. A quantitative core-loss EELS study was also performed to gain insight on the molecular structure of monolayer g-C_3_N_4_, as shown in Fig. 5b. The *sp*^2^-hybridized carbon and nitrogen atoms were expected for both the samples, g-C_3_N_4_–b and g-C_3_N_4_–m, and therefore, the bands could be rationalized as the 1* s*–π* and 1* s*–σ* transitions for both elements [13]. The atomic ratios (N/C) for g-C_3_N_4_–b and g-C_3_N_4_–m were also calculated from the EELS data as 1.26 for the former and 1.32 for the latter, respectively, which were fairly consistent with the theoretical one of 1.33 (Table S2). We have also compared the high-resolution XPS of g-C_3_N_4_–m and g-C_3_N_4_–b to understand how layer stacking could alter the C1s and N1s electron binding energies, as shown in Fig. S8 and found that all the binding energy data were in good agreement with the values reported for g-C_3_N_4_ [32]. The monolayer g-C_3_N_4_–m, on the other hand, gave the spectra, where both C1s and N1s peaks were shifted by 0.2 eV toward higher binding energies, due to the slightly shorter C–N bond, resulting in a hardening of the photoelectron mode [33]. One thing to note here is that the spectral intensities for g-C_3_N_4_–m became weaker than those for the bulk under the same condition, because of the reduction in the number of atoms generating XPS signals for the monolayer. The electronic structure assignments identified by EELS and XPS were also supported by the FT-IR spectra, as shown in Fig. 5c. The spectrum for the bulk phase (g-C_3_N_4_–b) showed the absorption peaks at 1638 and 1568 cm^−1^ ascribed to the C = N stretching vibrations, while the bands at 1410, 1320, and 1240 cm^−1^ were assigned as the aromatic C–N stretching vibrations [6]. However, the bands for the g-C_3_N_4_–m phase at 1568, 1410, 1320 and 1240 cm^−1^ were shifted to higher energy by 30, 2, 10 and 20 cm^−1^, respectively, compared to those for the bulk phase. The observed blue-shift for the monolayer was surely due to the disappearance of interlayer van der Waals interaction, which resulted in shorter *in-plane* bond distance, and eventually in higher force constants.

### UV–vis DRS and PL Analyses

**Fig. 6:**
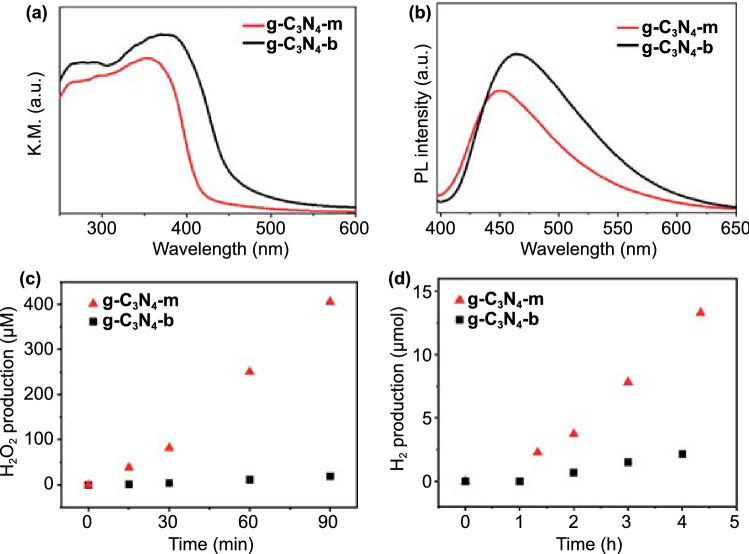
**a** UV–vis DRS and **b** PL spectra. Visible light (*λ* > 420 nm)-induced photocatalytic activity for **c** H_2_O_2_ generation and **d** H_2_ generation

To cross-confirm the formation of g-C_3_N_4_–m, its electronic band structure was compared to that of the bulk phase (g-C_3_N_4_–b), by analyzing the UV–vis DRS and PL data, as shown in Fig. 6a, b, respectively. The absorption edge in the UV–vis DRS showed a definitive blue shift from 470 (g-C_3_N_4_–b) to 419 (g-C_3_N_4_–m) nm, due to an increasing band gap from 2.64 to 2.96 eV. The band gap value for the monolayer is in a good agreement with the calculated value (3.03 eV) based on the Heyd–Scuseria–Ernzerhof (HSE) computation, reflecting the quantum size effect on electronic and optical properties of g-C_3_N_4_ [28]. Furthermore, the PL spectrum of g-C_3_N_4_–m also exhibited a blue shift of ∼30 nm compared to that of g-C_3_N_4_–b. Such a blue shift in PL spectra could also be rationalized by the quantum confinement effect resulting in the shift of conduction and valence bands to opposite directions [30]. The optical and electronic properties for typical 2D materials with the in-plane lattice structure seemed to be, in general, not changed from the bulk one, but those for the isolated monolayer, however, could be changed due to the electronic decoupling between adjacent layers [23], resulting in stronger in-plane bonding. One thing to underline here was the PL intensity, which was determined to be notably weaker for g-C_3_N_4_–m than for g-C_3_N_4_–b, clarifying the depression of charge recombination by the exfoliation into monolayer with efficient charge separation behavior (Fig. 6b). Such an increase of the lifetime of photoexcited electrons and holes was supposed to be effective in improving the photocatalytic activity of g-C_3_N_4_ [34]. All the present topographic and optical measurements proved our hypothesis that atomically thin monolayer of g-C_3_N_4_–m in the 2D limit was indeed isolated.

### Photocatalytic and Electrocatalytic Activity Measurements

The effect of monolayer formation on the photocatalytic activity of g-C_3_N_4_ was investigated by employing g-C_3_N_4_–m as photocatalysts for visible light (*λ* > 420 nm)-induced H_2_O_2_ and H_2_ generation. Even with the increase of bandgap energy upon the exfoliation, the g-C_3_N_4_–m displays much higher visible light photocatalytic activity for H_2_O_2_ generation than that of bulk g-C_3_N_4_–b, clearly demonstrating the beneficial effect of monolayer formation (Fig. 6c). The photocatalytic formation rate of H_2_O_2_ on g-C_3_N_4_–m was determined to be twenty-times faster than that on g-C_3_N_4_–b. In addition, for comparison, multilayered g-C_3_N_4_ nanosheets (described as g-C_3_N_4_–n hereafter) were also prepared by ultrasonication-assisted liquid exfoliation of bulk g-C_3_N_4_. The prepared g-C_3_N_4_-n was determined to have a basal thickness of around 2 nm (~ 6 layers, Fig. S9). We have also demonstrated that the present g-C_3_N_4_–m monolayer particles showed excellent photocatalytic properties compared to the g-C_3_N_4_–n, as shown in Fig. S10. The observed photocatalytic activity of g-C_3_N_4_–m for H_2_O_2_ generation was found to be the most efficient compared to the ever-reported from pure g-C_3_N_4_ materials (Table 1). Such a beneficial effect of monolayer on the photocatalyst performance was further evidenced by the visible light photocatalytic H_2_ evolution study. As presented in Fig. 6d, the photocatalytic efficiency of g-C_3_N_4_–m for visible light-driven H_2_ production was six times higher than that of g-C_3_N_4_–b. Such an unusual high efficiency in photocatalyst performance could be rationalized not only due to a remarkable increase of surface active sites, but also due to a significant enhancement of charge separation on the surface of semiconducting g-C_3_N_4_–m, as evidenced by the PL results (Fig. 6b). The present two step synthetic route to g-C_3_N_4_–m monolayer particles should be an ideal way of providing an economically feasible and ecofriendly methodology for the scalable production of H_2_O_2_.

**Table 1:** Comparison of the photocatalytic H_2_O_2_ generation of g-C_3_N_4_

g-C_3_N_4_	Experimental condition	Light source	H_2_O_2_ generation (μmol g^−1^ h^−1^)	References
None	9/1 (v/v) propan-2-ol/water (5 mL); 4 g L^−1^	2000 W Xe lamp *λ* > 420 nm); O_2_	13	[35]
None	9/1 (v/v) ethanol/water (5 mL); 4 g L^−1^	2000 W Xe lamp *λ* > 420 nm); O_2_	63	[35]
None	9/1 (v/v) butan-1-ol/water (5 mL); 4 g L^−1^	2000 W Xe lamp *λ* > 420 nm); O_2_	38	[35]
Surface defects	9/1 (v/v) ethanol/water (5 mL); 4 g L^−1^	2000 W Xe lamp *λ* > 420 nm); O_2_	188	[37]
C vacancies	water (100 mL); 1 g L^−1^	300 W Xe lamp *λ* > 420 nm); O_2_	90	[37]
N vacancies	water (100 mL); 1 g L^−1^	300 W Xe lamp *λ* > 420 nm); O_2_	15	[37]
N vacancies	20% (v) propan-2-ol/water (60 mL); 0.83 g L^−1^	solar simulator *λ* > 420 nm); O_2_	97	[38]
N vacancies	water (100 mL); 1.0 g L^−1^	300 W Xe lamp *λ* > 420 nm); O_2_	300	[39]
None	5/95 (v/v) methanol/water (100 mL); 0.5 g L^−1^	300 W Xe lamp *λ* > 420 nm); O_2_	25	This work
Nanosheet	5/95 (v/v) methanol/water (100 mL); 0.5 g L^−1^	300 W Xe lamp *λ* > 420 nm); O_2_	208	This work
None	5/95 (v/v) methanol/water (100 mL); 0.5 g L^−1^	300 W Xe lamp *λ* > 420 nm); O_2_	540	This work

**Fig. 7:**
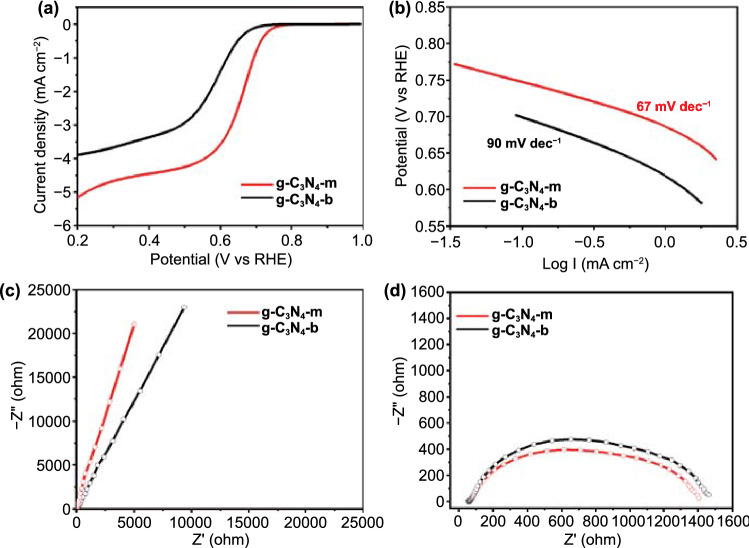
**a** Linear sweep voltammograms (LSV) curves of ORR. **b** Tafel plots, and Nyquist plots measured at **c** OCV and **d** 0.6 V

In addition to the photocatalyst application, monolayer g-C_3_N_4_–m was also tested as ORR electrocatalyst. As plotted in Fig. 7a, g-C3N4–m showed relatively higher positive on-set potential of 0.76 V at 0.1 mA cm^−2^ than that of g-C_3_N_4_–b (0.66 V), clearly demonstrating that the monolayer formation resulted in the improved ORR property. To understand the morphological effect of g-C_3_N_4_ on the ORR electro-catalysis kinetics, the Tafel slopes were calculated for the present g-C_3_N_4_–m and g-C_3_N_4_–b, as shown in Fig. 7b. The former exhibited a much smaller Tafel slope of 67 mV dec^−1^ than that of g-C_3_N_4_–b (90 mV dec^−1^), emphasizing again the usefulness of g-C_3_N_4_–m in promoting the ORR kinetics [33]. Such a fast reaction kinetics of g-C_3_N_4_–m responsible for its improved ORR activity could be rationalized by the fact that the monolayer formation resulted in the effective exposure of surface active sites. In addition, the influence of crystal morphology on charge transfer behavior was probed by measuring the EIS data. As presented in Fig. 7c, the present g-C_3_N_4_ materials commonly display a straight line of Warburg region at OCV. The slope of Z_re_ vs *ω*^−1/2^ plot allows to determine the Warburg coefficient (*σ*_w_), which is inversely proportional to ion diffusion coefficient on the electrode [34]. The *σ*_w_ value determined was 6460 for g-C_3_N_4_–m and 12,101 Ω s^−0.5^ for g-C_3_N_4_–b, respectively. Such a smaller *σ*_w_ value for g-C_3_N_4_–m compared to the bulk indicated the remarkable improvement of ion polarizability in the monolayer. At an applied potential of 0.6 V, the similar Nyquist plot composed of a semicircle in high- and mid-frequency regions was discernible for the monolayer and the bulk as shown in Fig. 7d. The radius of semicircle appeared to be smaller for g-C_3_N_4_–m than for g-C_3_N_4_–b. Since the radius of this semicircle is inversely proportional to the charge transfer resistance (*R*_ct_) [37], the present EIS results provided again a clear evidence for the improvement in interfacial charge transfer kinetics upon the formation of monolayer, which resulted in an additional contribution to the improved ORR activity of g-C_3_N_4_–m. Surprisingly, the present g-C_3_N_4_–m monolayer particles showed excellent photo and electrocatalytic properties compared to the g-C_3_N_4_ nanosheets (g-C_3_N_4_–n), though their catalytic activities for ORR were more or less lower than that of commercial Pt/C, as shown in Figs. S9-S11.

## Conclusions

In conclusion, g-C_3_N_4_–m in the perfect 2D limit was successfully realized by the well-defined chemical strategy based on the 2D Mica template route; in the first step, cyanamide precursor molecules were intercalated and polymerized in two dimensionally confined nanospace (Mica) to form g-C_3_N_4_–m–Mica hybrid on the basis of IPR. Then, in the second step, selective etching of 2D template was made in an acidic solution to separate the g-C_3_N_4_–m nanoflakes from the g-C_3_N_4_–m–Mica hybrid. Thus, obtained colloidal suspension containing g-C_3_N_4_–m was found to be stable for several weeks, due to its positive zeta potential of + 37 mV. And the formation of g-C_3_N_4_–m was evidenced by comparing the UV–vis, DRS and PL spectra of the monolayer with those of the bulk. Stoichiometry and chemical structures of the tri-s-triazine unit were confirmed by XPS and EELS spectra for the monolayer and the bulk. The most striking evidence was made from Cs-HRTEM measurements by observing directly the atomic structures of g-C_3_N_4_ unit cell, which was again supported by the corresponding HRTEM image simulation results. We, therefore, report here that a long-standing problem to realize atomic monolayers of g-C_3_N_4_ is now solved by a well-defined chemical strategy. The difficulty in exfoliating such a 2D material like g-C_3_N_4_ without stripping off layer by layer with scotch tape can be overcome by the proof of concept study based on the well-defined chemical strategy not previously developed. From the viewpoint of practical application, we successfully demonstrated that the present g-C_3_N_4_–m showed outstanding photocatalytic activity for H_2_O_2_ generation as well as excellent electrocatalytic activity for ORR. Of prime importance is that the present g-C_3_N_4_ monolayer shows the highest efficiency for visible light-induced H_2_O_2_ production ever published among any pure g-C_3_N_4_ nanomaterials [35–42], emphasizing the crucial role of monolayer morphology in optimizing the catalyst performance of g-C_3_N_4_.

## Supplementary Information

Below is the link to the electronic supplementary material.

Supplementary file1 (PDF 1435 kb)
