# Supplementary material for: Monolayer Graphitic Carbon Nitride as Metal-Free Catalyst with Enhanced Performance in Photo- and Electro-Catalysis
Source: Nanomicro Lett. 2022 Feb 3;14:55. doi: 10.1007/s40820-022-00794-9 (PMC8814173; doi:10.1007/s40820-022-00794-9)
Supplement: Supplementary file 1 — Supplementary file1 (PDF 1435 kb) [file 40820_2022_794_MOESM1_ESM.pdf]

Supporting Information for

Monolayer Graphitic Carbon Nitride as Metal-Free Catalyst with Enhanced Performance in Photo- and Electro-Catalysis

Huiyan Piao[1], Goeun Choi[1, 2, 3], Xiaoyan Jin[4], Seong-Ju Hwang[4], Young Jae Song[5, 6], Sung-Pyo Cho[7, 8,] *, and Jin-Ho Choy[1, 9, 10,] *

[1]Intelligent Nanohybrid Materials Laboratory (INML), Institute of Tissue Regeneration Engineering (ITREN), Dankook University, Cheonan 31116, Republic of Korea

[2]College of Science and Technology, Dankook University, Cheonan 31116, Republic of Korea

[3]Department of Nanobiomedical Science and BK21 PLUS NBM Global Research Center for Regenerative Medicine, Dankook University, Cheonan 31116, Republic of Korea

[4]Department of Materials Science and Engineering, College of Engineering, Yonsei University, Seoul 03722, Republic of Korea

[5]SKKU Advanced Institute of Nanotechnology (SAINT), Sungkyunkwan University (SKKU), Suwon 440-746, Republic of Korea

[6]Department of Nano Engineering, Sungkyunkwan University (SKKU), Suwon 440-746, Republic of Korea

[7]National Center for Inter-University Research Facilities (NCIRF), Seoul National University, Seoul 08826, Republic of Korea

[8]Graphene Research Center, Advanced Institute of Convergence Technology, Suwon 16229, Republic of Korea

[9]Department of Pre-medical Course, College of Medicine, Dankook University, Cheonan 31116, Republic of Korea

[10]Tokyo Tech World Research Hub Initiative (WRHI), Institute of Innovative Research, Tokyo Institute of Technology, Yokohama 226-8503, Japan

*Corresponding authors. E-mail: chosp@snu.ac.kr (Sung-Pyo Cho); jhchoy@dankook.ac.kr (Jin-Ho Choy)

Supplementary Tables and Figures

Table S1 Summary of tri-s-triazine-based g-C_3N_4 nanosheets and monolayer. Synthetic methods and characterization are listed

**Precursor**	**Method**	**Thickness**	**Characterization (Morphology)**	**Characterization (XRD)**	**Compare with bulk g-C_3N_4**		**References**
					**FT-IR**	**XPS**	
Dicyanamide	Bottom up	0.4-0.5	None	Crystallinity	Same	Same	[S1]
Dicyanamide	Bottom up	0.4	TEM (nanosheet image)	Crystallinity	Same	Same	[S2]
Melamine	Bottom up	0.38	SEM, TEM (nanosheet image)	Crystallinity	Same	Same	[S3]

Melamine	Bottom up	0.5	SEM, TEM (nanosheet image)	Crystallinity	Same	Same	[S4]
Melamine	Bottom up	0.64	SEM, HRTEM (nanosheet image)	Crystallinity	Same	Same	[S5]
Urea	Bottom up	0.6	TEM (nanosheet image)	Crystallinity	Same	Same	[S6]
Melamine	Bottom up	0.32	SEM, TEM (nanosheet image)	Crystallinity	Same	Same	[S7]
Urea	Bottom up	0.34	SEM, TEM (nanosheet image)	Crystallinity	Same	Same	[S8]
Cyanamide	Top down	0.45	Cs-HRTEM (atomic image)	Amorphous	Blue shift	Blue shift	This work

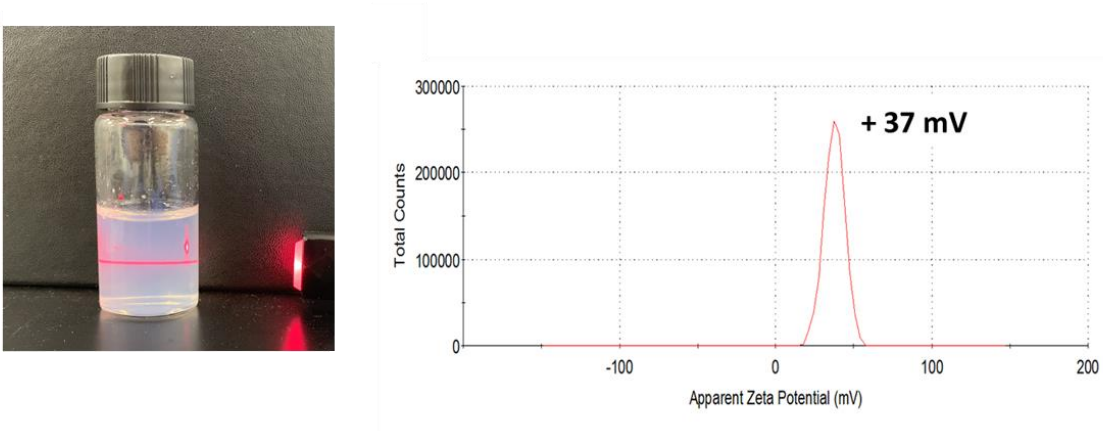

Fig. S1 Tyndall effect and zeta potential of the g-C_3N_4–m colloidal suspension.

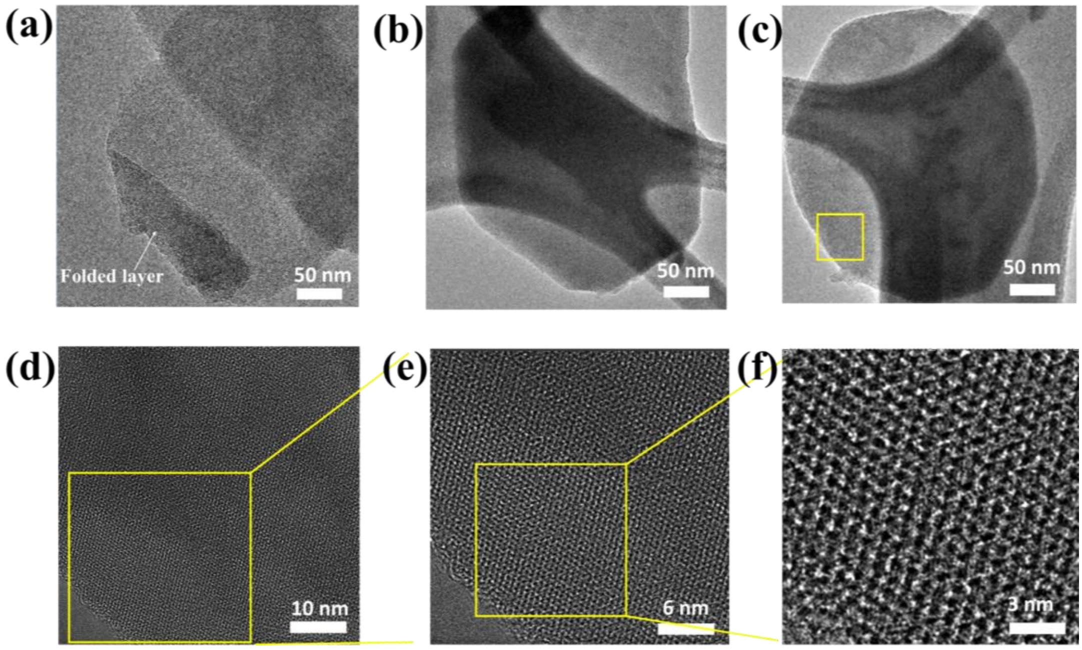

Fig. S2 Typical high resolution-transmission electron microscopy (HR-TEM) images of g-C_3N_4–m. **a-c** Transmission electron microscopy (TEM) images of randomly chosen g-C_3N_4–m. **d-f** A magnified view of the area marked by the yellow box in c-e, respectively. The g-C_3N_4–m exhibited well-known orderly structure with hexagonal symmetry along with triangular cavity formed by encirclement of three adjacent heptazine units on the surface

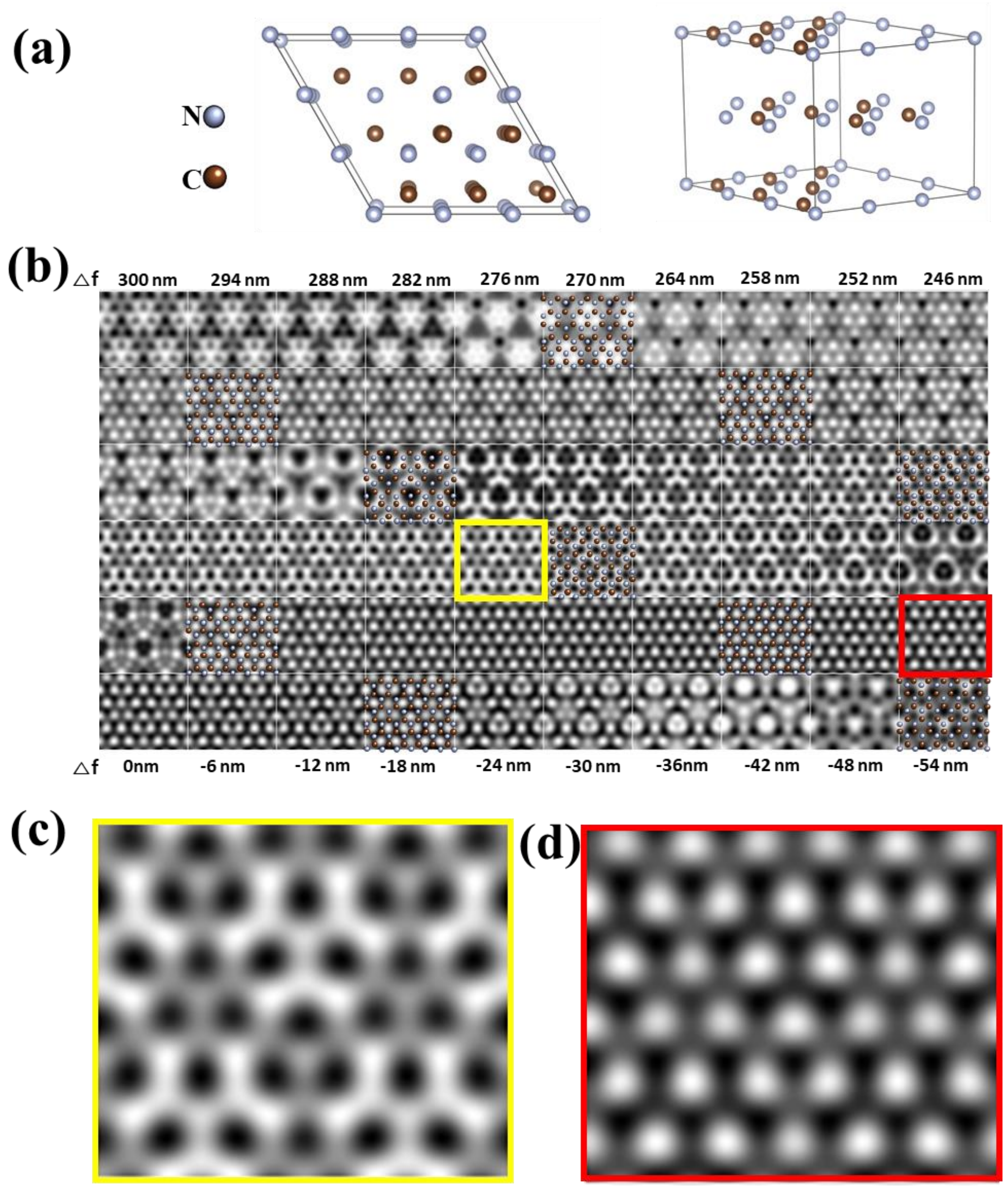

Fig. S3 Unit cell structures with the simulated through-focal series spherical aberration (Cs) corrected high-resolution transmission electron microscopy (Cs-HRTEM) images for g-C_3N_4–b. **a** Top view of super unit cell and side view of unit cell. **b** The simulated through-focal series showing the effect of objective lens defocus on image contrast and the corresponding atomic identification. The simulation program operated was MacTempas. (the acceleration voltage of 80 kV, Cs of 0.034 mm, the spread of defocus of 30 nm, the beam convergence of 2 mrad, the specimen thickness of 1 nm and the defocus value of a step of 6 nm in the range from 54 to 300 nm were used for the simulation. **c, d** Magnified images of the areas marked by the yellow (f = 90 nm) and red boxes (f = 0 nm) in **b**

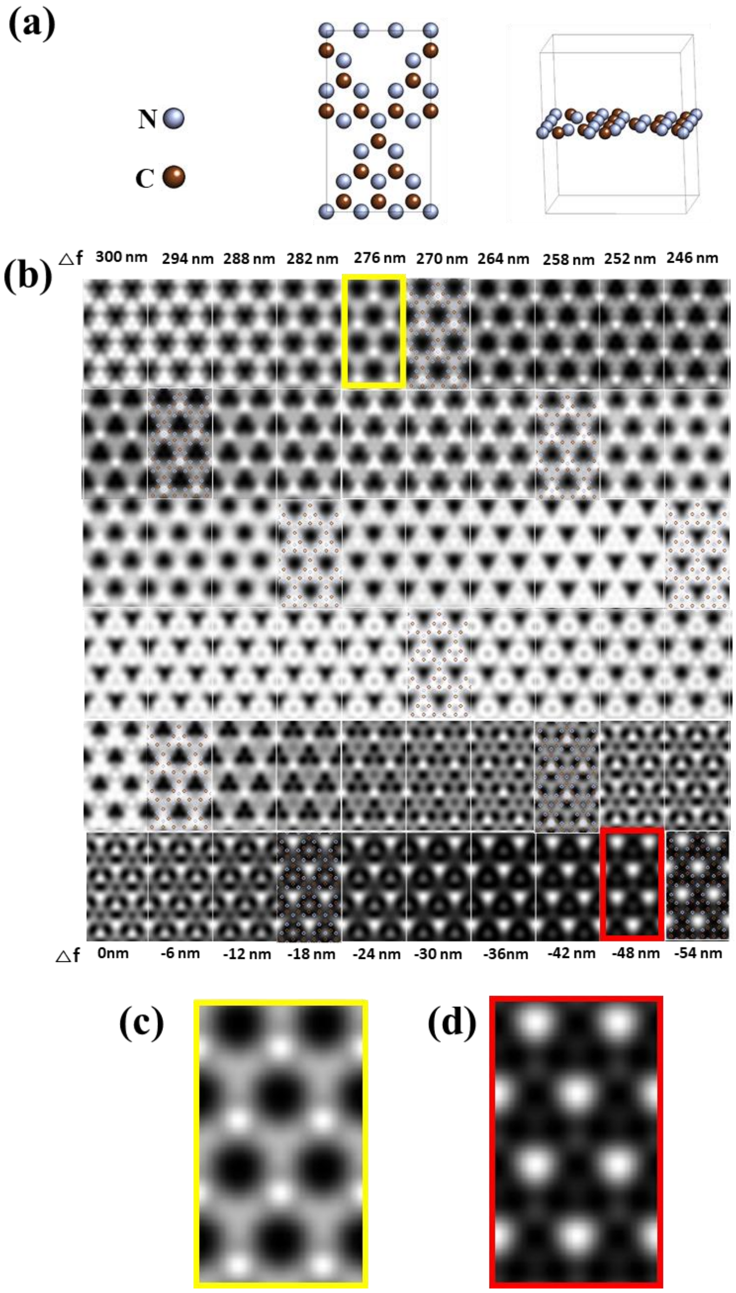

Fig. S4 Unit cell structures with the simulated through-focal series Cs-HRTEM images for g-C_3N_4–m. **a** Top view of super unit cell and side view of unit cell. **b** The through-focal series simulation showing the effect of objective lens defocus on contrast in images and the corresponding atomic identification. The simulation program was MacTempas. The acceleration voltage of 80 kV, Cs of 0.034 mm, the spread of defocus of 30 nm, the beam convergence of 2 mrad, the specimen thickness 1 nm and the defocus value of a step of 6 nm in the range from 54 to 300 nm were used for the simulation. **c, d** Magnified images of the areas marked by the yellow (f = 276 nm) and red (f = 48 nm) boxes in b, respectively

In general, atomically thin and freely suspended 2D supramolecular entities, such as the monolayers of g-C_3N_4-m in solution as shown in this study, are not expected to be perfectly flat; i.e. they are rather flexible and probably exhibit a microscopic corrugation or a randomly curved single molecular sheet. Such a microscopic crumpling has often raised a problem in detailed HRTEM analysis, since the phase contrast seems to be extremely sensitive particularly for 2D compounds to defocus (*f*), spherical aberration (Cs) and alignment conditions of the

objective lens, which means that there are often multiple defocus values at different microscopic corrugating positions, even within the same HRTEM image. In this study, the defocus series of simulated images were performed to assist in interpreting experimental Cs-HRTEM images. Under a certain condition, for instance, with 276 nm defocus, the white dots are coinciding with the atom positions as shown in Fig. S4c, but in the case with 48 nm defocus, a reversed contrast can be seen as well demonstrated in Fig. S4d. After performing detailed simulation studies, we were able to find a systematically periodic structure with a triangle arrangement of bright white dots and a triangular cavity in the hexagonal, which is well consistent with the structure model shown in Fig. S4a.

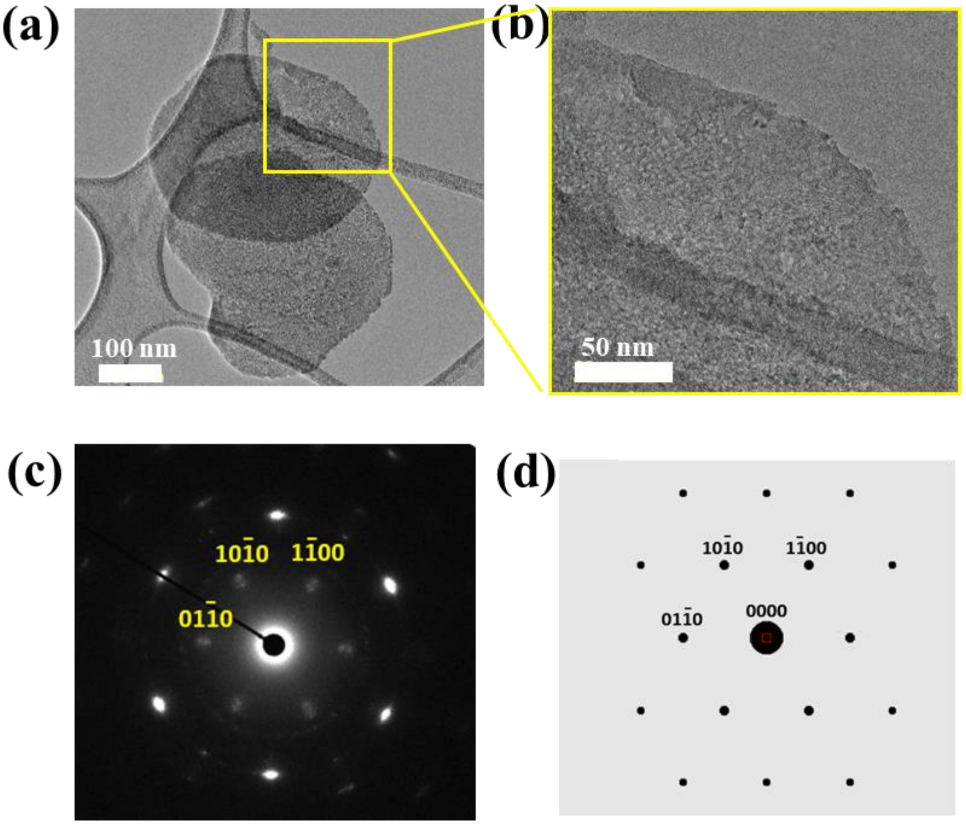

Fig. S5 Selected area electron diffraction (SAED) patterns of g-C_3N_4–b. **a** A low-magnification TEM image of g-C_3N_4–b. **b** Cs-HRTEM image which is magnified from the yellow box in a. **c** The experimental diffraction pattern and **d** the simulated one. The electron beam is given along the [*0001*] g-C_3N_4–b axis

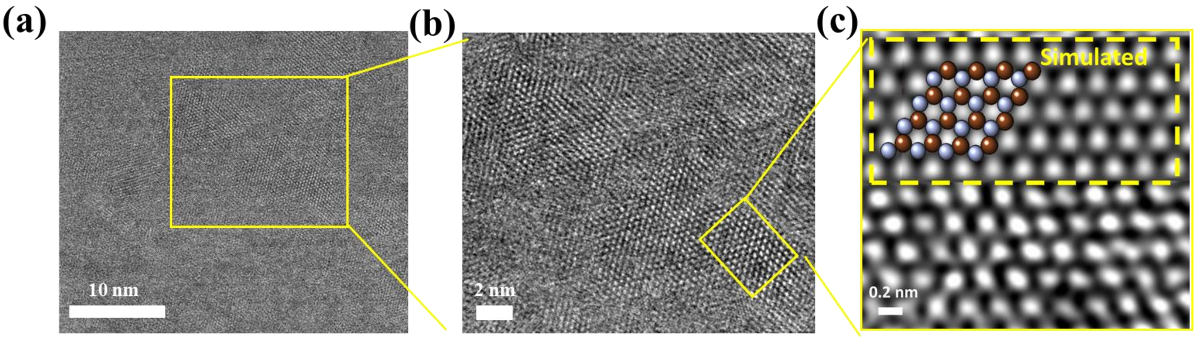

Fig. S6 Cs-HRTEM images of g-C_3N_4–b. **a** Low-magnification TEM image of g-C_3N_4–b. **b** The image magnified from the yellow box in a. **c** A Wiener-filtered Cs-HRTEM image experimentally observed, which is magnified from the yellow box in b. And the inset is theoretically simulated image with the corresponding atomic model overlaid for g-C_3N_4-b

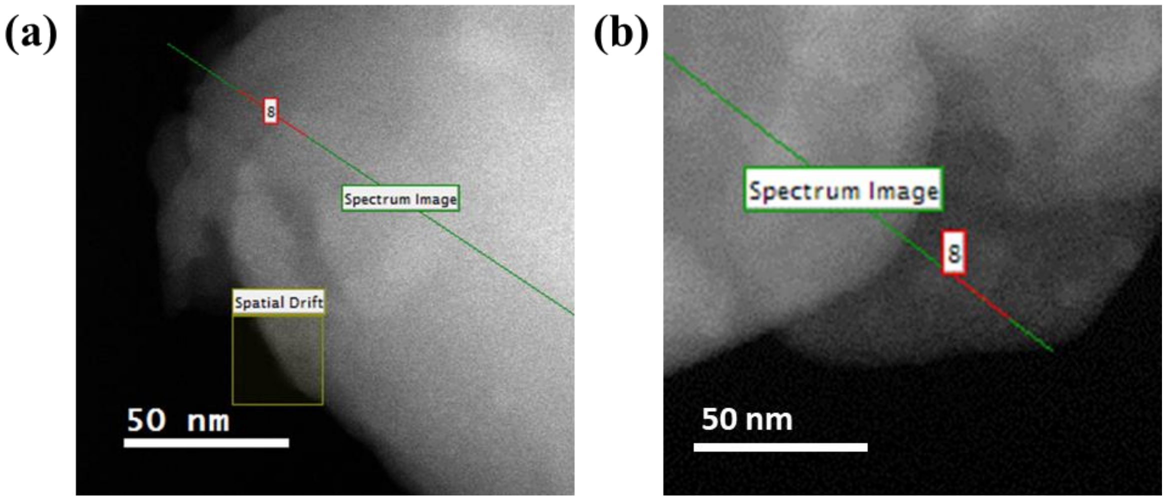

Fig. S7 Annular dark field scanning transmission electron microscopy (ADF-STEM) images of **a** g-C_3N_4–b and **b** g-C_3N_4–m were marked with red color-coded lines for the corresponding electron energy loss spectroscopy (EELS) data

Table S2 Elemental analysis of g-C_3N_4–b and g-C_3N_4–m determined from their EELS spectra

Element	Atomic % Theoretical	g-C_3N_4–b Experimental	g-C_3N_4–m Experimental
**C**	42.9	44.2	43.2
**N**	57.1	55.8	56.8
**N/C Ratio**	1.33	1.26	1.32

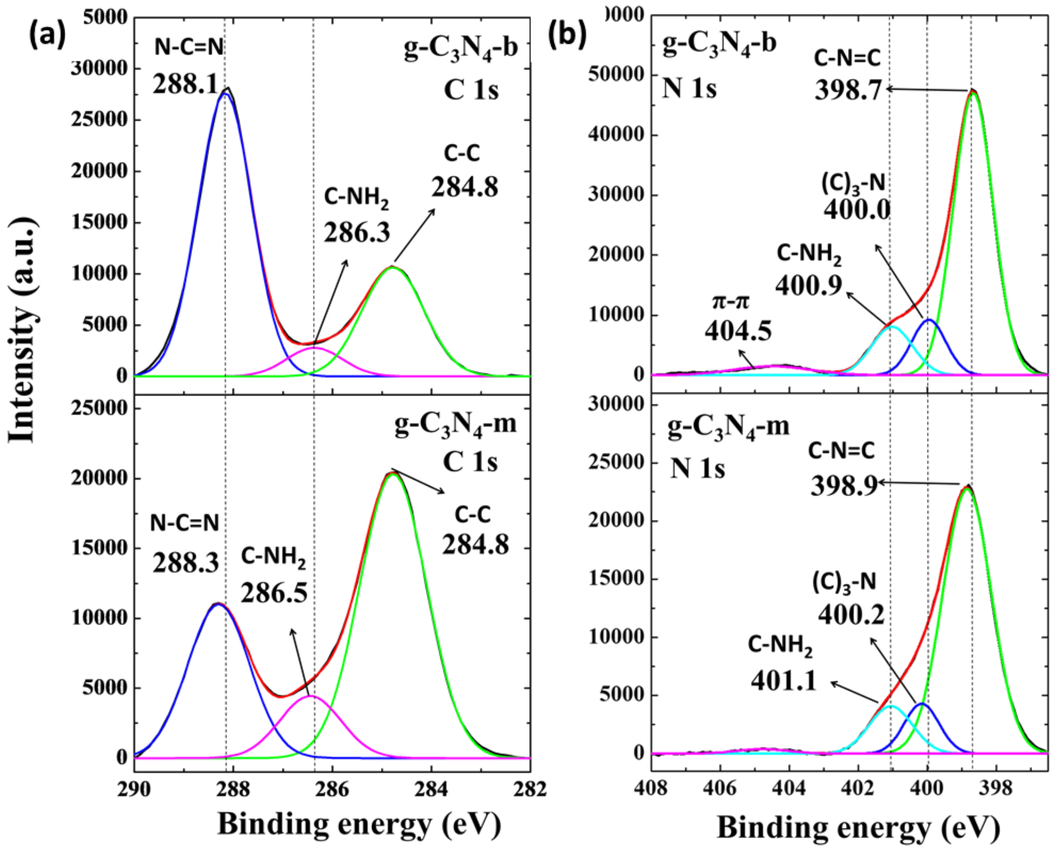

Fig. S8 X-ray photoelectron spectra (XPS) of **a** C1s and **b** N1s for g-C_3N_4–b and g-C_3N_4–m

The C 1s peak here for the bulk could be deconvoluted into three peaks at 284.8, 286.3 and 288.1 eV, corresponding to the signals from the standard reference carbon, the carbon atoms in C-NH_2 and the sp^2-hybridized carbon in the aromatic ring (N-C=N), respectively [S9]. The binding energy for the N1s spectrum was also deconvoluted into three peaks, 398.7, 400.0 and 400.9 eV, corresponding to the sp^2-hybridzed aromatic N bonded to carbon atoms (C-N=C), tertiary N bonded to carbon atoms in the form of N-(C_3), and to the quaternary N bonded to three carbon atoms in the aromatic rings (C-NH_2), respectively [S10]. An additional small peak at 404.5 eV was ascribed to the π-π excitation occurring between layers [S3].

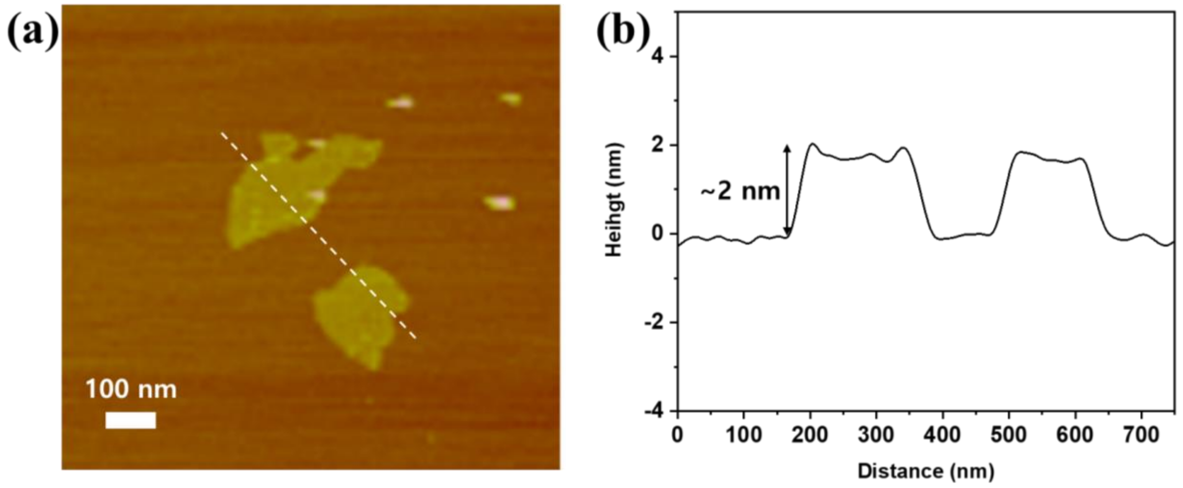

Fig. S9 Atomic force microscope (AFM) image of g-C_3N_4-n. **a** The g-C_3N_4–n dispersed on the freshly-cleaved muscovite mica substrate. **b** The height profile along the white dashed line in a

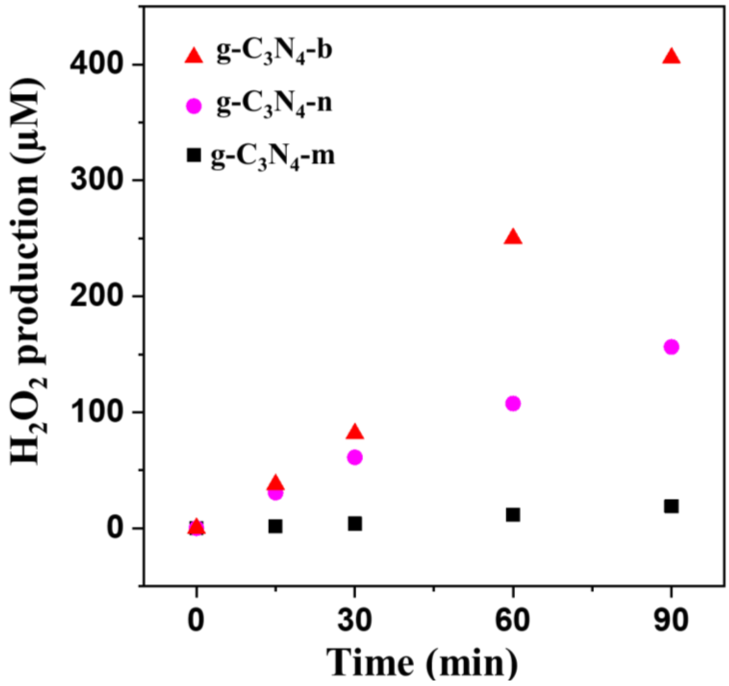

Fig. S10 Photocatalytic activity for visible light-induced H_2O_2 generation

The excellent photocatalytic property of g-C_3N_4–m was also evidenced by the visible light (λ> 420 nm)-induced generation test of H_2O_2, and the photocatalytic formation rate of H_2O_2 on g-C_3N_4-m was determined to be twenty times and three times faster than that on g-C_3N_4–b and g-C_3N_4–n

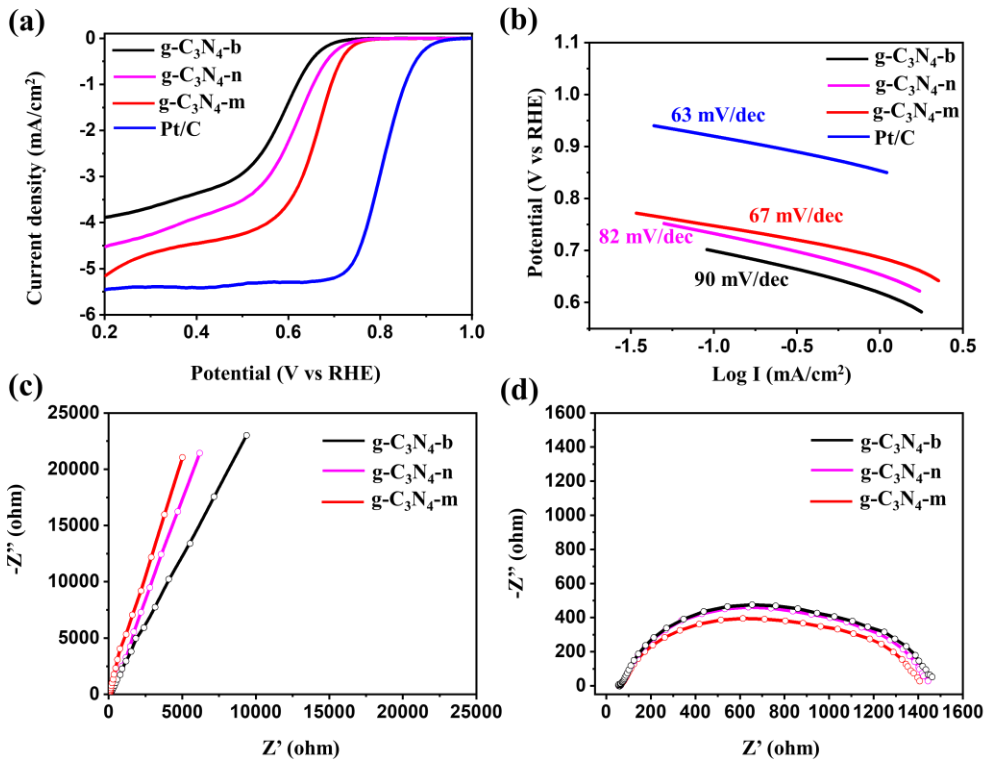

Fig. S11 a Linear sweep voltammograms (LSV) curves of ORR, **b** Tafel plots, and Nyquist plots measured at **c** OCV and **d** 0.6 V

The monolayer g-C_3N_4–m showed excellent electro-catalytic properties compared to the bulk and the nanosheets, due to the fact that the formation of monolayers resulted in a remarkable increase in active sites.

Supplementary References

[1] H. Zhao, H. Yu, X. Quan, S. Chen, H. Zhao et al., Atomic single layer graphitic-C_3N_4: fabrication and its high photocatalytic performance under visible light irradiation. RSC Adv. **4**(2), 624-628 (2014). https://doi.org/10.1039/C3RA45776A

[2] J. Xu, L. Zhang, R. Shi, Y. Zhu, Chemical exfoliation of graphitic carbon nitride for efficient heterogeneous photocatalysis. J. Mater. Chem. A **1**(46), 14766-14772 (2013). https://doi.org/10.1039/c3ta13188b

[3] Q. Lin, L. Li, S. Liang, M. Liu, J. Bi et al., Efficient synthesis of monolayer carbon nitride 2D nanosheet with tunable concentration and enhanced visible-light photocatalytic activities. Appl. Catal. B Environ. **163**, 135-142 (2015). https://doi.org/10.1016/j.apcatb.2014.07.053

[4] H. Zhao, H. Yu, X. Quan, S. Chen, Y. Zhang, Fabrication of atomic single layer graphitic-C_3N_4 and its high performance of photocatalytic disinfection under visible light irradiation. Appl. Catal. B Environ. **152-153**, 46-50 (2014). https://doi.org/10.1016/j.apcatb.2014.01.023

[5] X. Shi, J. Wu, J. Zhong, H. Xu, Y. Yang et al., The photovoltaic and photoconductive photodetector based on GeSe/2D semiconductor van der Waals heterostructure. Appl. Phys. Lett. **116**(14), 138-146 (2016). https://doi.org/10.1063/1.5143961

[6] Q. Liang, Z. Li, Y. Bai, Z.H. Huang, F. Kang et al., Reduced-sized monolayer carbon nitride nanosheets for highly improved photoresponse for cell imaging and photocatalysis. Sci. China Mater. **60**, 109-118 (2017). https://doi.org/10.1007/s40843-016-5131-9

[7] C. Rao, M. Xie, S. Liu, R. Chen, H. Su et al., Visible light-driven reforming of lignocellulose into H_2 by intrinsic monolayer carbon Nitride. ACS Appl. Mater. Interfaces **13**(37), 4423-4453 (2021). https://doi.org/10.1021/acsami.1c10842

[8] L. Jiang, J. Li, K. Wang, G. Zhang, Y. Li et al., Low boiling point solvent mediated strategy to synthesize functionalized monolayer carbon nitride for superior photocatalytic hydrogen evolution. Appl. Catal. B Environ. **260**, 118181 (2020). https://doi.org/10.1016/j.apcatb.2019.118181

[9] G.P. Mane, S.N. Talapaneni, K.S. Lakhi, H. Ilbeygi, U. Ravon et al., Highly ordered nitrogen-rich mesoporous carbon nitrides and their superior performance for sensing and photocatalytic hydrogen generation. Angew. Chem. Int. Ed. **56**(29), 8481-8485 (2017). https://doi.org/10.1002/anie.201702386

[10] G. Constantinescu, A. Kuc, T. Heine, Stacking in bulk and bilayer hexagonal boron nitride. Phys. Rev. Lett. **111**, 036104 (2013). https://doi.org/10.1103/PhysRevLett.111.036104
